# Patients with positive HER-2 amplification advanced gastroesophageal junction cancer achieved complete response with combined chemotherapy of AK104/cadonilimab (PD-1/CTLA-4 bispecific): A case report

**DOI:** 10.3389/fimmu.2022.1049518

**Published:** 2022-12-08

**Authors:** Jieqiong Peng, Qiang Zhu, Ziru Peng, Zhen Chen, Yuantao Liu, Bo Liu

**Affiliations:** ^1^ Department of Oncology, Shandong Cancer Hospital and Institute, Shandong First Medical University and Shandong Academy of Medical Sciences, Jinan, China; ^2^ Department of Clinical Laboratory, Sishui County People’s Hospital, Sishui, China; ^3^ Department of Pathology, Linyi Cancer Hospital, Linyi, China; ^4^ Medical Department, Nanjing Geneseeq Technology Inc., Nanjing, Jiangsu, China

**Keywords:** PD-1/CTLA-4 bispecific, AK104/cadonilimab, HER-2 positive, complete response, advanced gastroesophageal junction cancer

## Abstract

**Background:**

Human epidermal growth factor receptor 2 (HER2) is the most prominent therapeutic target for advanced gastric (G)/GEJ cancer. However, targeted therapy did not significantly improve survival. Currently, there are no regimens for the treatment of HER-2 amplification that exclude targeted agents.

**Case presentation:**

A 42-year-old man was diagnosed with adenocarcinoma of GEJ (stage IV) with liver metastasis and lung metastasis. The patient was enrolled in a trial that excluded patients with known HER2-positivity: AK104, a PD-1/CTLA-4 bispecific antibody, combined with chemotherapy (mXELOX) as first-line therapy for advanced gastric G/GEJ cancer (NCT03852251). After six cycles of AK104 combined with chemotherapy therapy, immune-related pulmonary toxicity was observed. We rechallenged AK104 after hormone therapy, and no further pulmonary toxicity was observed. Immune-related hepatitis occurred in the patient during immunotherapy combined with single-drug capecitabine therapy. After combining steroid therapy with mycophenolate mofetil, the patient’s immune hepatitis improved. Nevertheless, the patient was excluded from the clinical study due to the long-term absence of medication. Antitumor therapy was also discontinued in view of the patient’s adverse immune response. The patient did not receive subsequent immune antitumor therapy, and immune-related hepatitis still occurred intermittently, but the disease evaluation was maintained at PR. A complete response was confirmed by PET/CT and the biopsy specimen from gastroscopy on 2020-06-10. Next generation sequencing of biopsy tissue was used to guide subsequent therapy at a recent follow-up visit. The results indicated that ERBB2 mutations occurred at copy number 58.4934 (HER-2), TMB = 3.1, MSS. IHC: EBV (−), PD-L1 CPS = 3, HER-2 (3+).

**Conclusion:**

Patients with HER-2-positive advanced GEJ cancer received PD-1/CTLA-4 bispecific immunotherapy combined with chemotherapy and achieved complete remission. It offers a novel, highly specific, and highly potent therapeutic option for HER-2-positive patients. Its use should be considered as a new treatment when trastuzumab is not viable. Currently, we are working to overcome this resistance.

## Introduction

HER-2 is the most prominent therapeutic target in advanced gastric (G) or gastroesophageal junction (GEJ) cancer (1). Since 2010, combination therapy with the anti-HER2 antibody trastuzumab and chemotherapy has become the standard first-line treatment for patients with HER-2-positive G/GEJ cancer (2). The development of a novel bispecific antibody that simultaneously binds to two distinct HER-2 epitopes (KN026) and the use of antibody–drug conjugates (ADC, such as T-DM1 and DS8201 and RC48) having a bystander effect are providing new tools to fight heterogeneity in HER-2 positive advanced cancer (3–5). Several studies have confirmed that anti-HER-2 effects involve antibody-dependent cell-mediated cytotoxicity by immune mechanisms superior to intracellular signaling (6). Immunotherapy plays an increasingly important role in the field of anti-tumor drugs and has achieved considerable clinical success. In the process of HER-2 negative advanced gastric cancer therapy, immune checkpoint inhibitors (natriculumab/sintilimab) combined with chemotherapy compared to pure chemotherapy for advanced G/GEJ First-line treatment of cancer has achieved overall survival (OS) and progression-free survival (PFS) benefits (7, 8). As indicated by the recent positive results of the KEYNOTE-811 trial, the immune effects of anti-HER-2 therapy can be better understood, and the effectiveness of the combination of immunotherapy and anti-HER2 therapy can be elucidated (9). This combination of immunologic targeting and chemotherapy has been recommended by the FDA. Currently, there are no regimens for the treatment of HER-2 amplification positivity that exclude targeted agents. We report a case of immune checkpoint inhibition combined with chemotherapy for the treatment of patients.

## Case report

**Figure 1:**
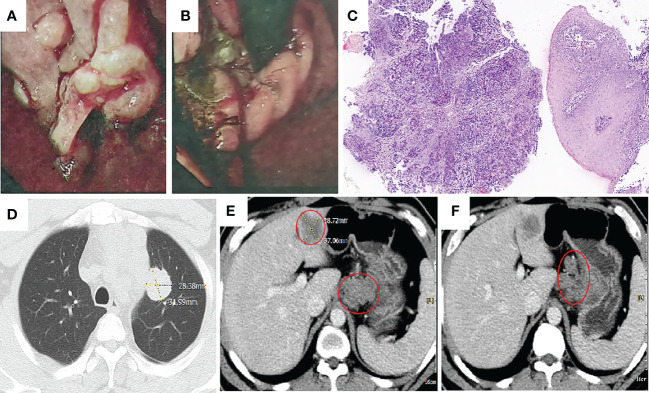
Imaging taken at baseline before initiation of treatment. **(A, B)** Gastroscopy illustrated lower esophagus, cardia and cardia by lumen narrowing. There is a huge ulcer in the cardia. The nodules at the bottom are uneven and covered with dirt moss. **(C)** Hemotoxylin and eosin (H&E): cardia with poorly differentiated adenocarcinoma (H&E, ×100 original magnification). **(D–F)** Computed tomography (CT) taken at the primary GEJ cancer and liver metastasis, lung metastasis and multiple lymph node metastasis.

A 42-year-old man has no clear incentive to present an eating obstruction in July 2020. Symptoms worsen when hard and dry foods are consumed, accompanied by paroxysms of dull pain in the upper left abdomen, no chest tightness or pain, no nausea and vomiting, no hematemesis and melena, no fever and chills, and other discomfort. No history of autoimmune disease, no pneumonia, interstitial lung disease, no chronic obstructive pulmonary disease (COPD), denial of hepatitis B virus (HBV) or hepatitis C virus (HCV), human immunodeficiency virus (HIV) carrier, no recent vaccinations. He visited a local hospital on 13 August 2020. Gastroscopy revealed the lower esophagus, cardia and cardia by lumen narrowing, allowing endoscopy to pass through. There is a huge ulcer in the cardia. The nodules at the bottom are uneven and covered with dirt moss (**Figures 1A, B**). Biopsy pathology: poorly differentiated adenocarcinoma (**Figure 1C**). The patient came to our hospital for further diagnosis and treatment 17 August 2020. Contrast-enhanced Computed tomography (CT) of the cervicothoracic abdomen and pelvis demonstrated: cardiac cancer involving the esophagus and lesser curvature of stomach, with multiple lymph node metastasis; superior lobe metastasis of the left lung; hepatic metastasis (**Figures 1D–F**). Eastern Oncology Collaborative Group (EOCG): 1, the patients had poor economic foundation, but as the breadwinner of the family, the patients and their families had a strong desire for therapy.

**Figure 2:**
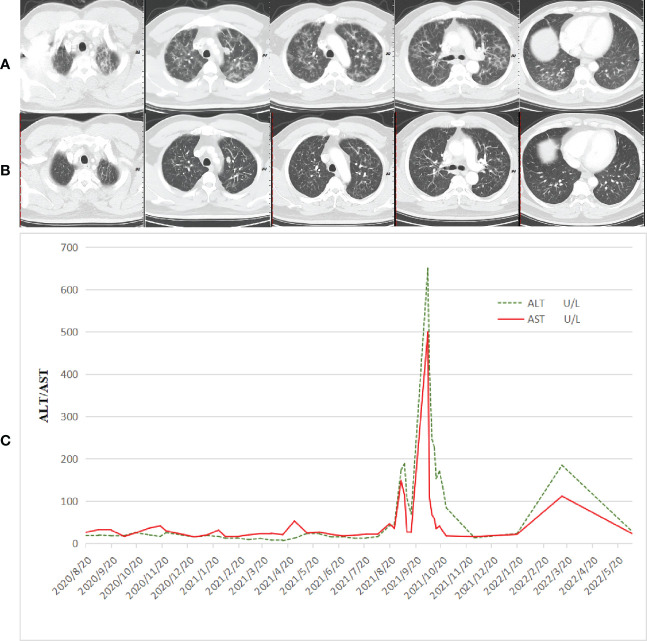
Adverse reactions that occurred during treatment. **(A)** CT taken at multiple floc and patchy high-density shadows in both lungs, appearance of interstitial pneumonia. **(B)** CT taken at the pneumonia was better than before, and the lung metastatic lesions continued PR. **(C)** Immune-related hepatitis occurred in the patient during immunotherapy combined with single-drug capecitabine therapy. After combined steroid therapy with mycophenolate mofetil, the patient’s immune hepatitis improved. The patient did not receive subsequent immune antitumor therapy, and immune-related hepatitis still occurred intermittently.

After communication with the patient and comprehensive consideration, the patient requested to be enrolled in the “open-label study of AK104 (PD-1/CTLA-4 bispecific antibody)” (10). Patients with unknown HER-2 status or negative results could be included in the group. He did not undergo HER-2 and PD-L1 tests at enrollment. Six cycles of AK104 + mXELOX/q14d (AK104 6 mg/kg d1+ oxaliplatin 85 mg/m^2^ d1 + capecitabine 1,000 mg/m^2^ d1–10/Q14d) were initiated on 27 August 2020. A partial response (PR) was assessed by CT after three and six cycles of treatment (primary foci and hepatic and lung metastatic lesions were markedly decreased). After the sixth treatment cycle, the patient showed symptoms of fatigue, wheezing after activity, palpitation, cough, phlegm, dry mouth, and loss of appetite. On 15 November 2020, general bacterial sputum culture and identification were performed. No bacteria associated with inflammation were identified. Detection of 13 respiratory pathogens: hemophilus influenzae positive. PCT: 0.10 ng/ml. Chest CT: multiple floc and patchy high-density shadows in both lungs, appearance of interstitial pneumonia (**Figure 2A**). He had not caught a cold recently and had no symptoms of fever. In addition, symptoms and additional examinations were combined to rule out the virus/bacterial pneumonia, considering the possibility of immune pneumonia. Antitumor therapy was interrupted, methylprednisolone sodium succinate (MPSS) 80 mg iv drip for 5 days, oral prednisone acetate tablets (taper off), and the patient’s symptoms were markedly improved. A CT scan performed on 28 December 2020 showed that the pneumonia was better than before, and the lung metastatic lesions continued PR (**Figure 2B**).

**Figure 3:**
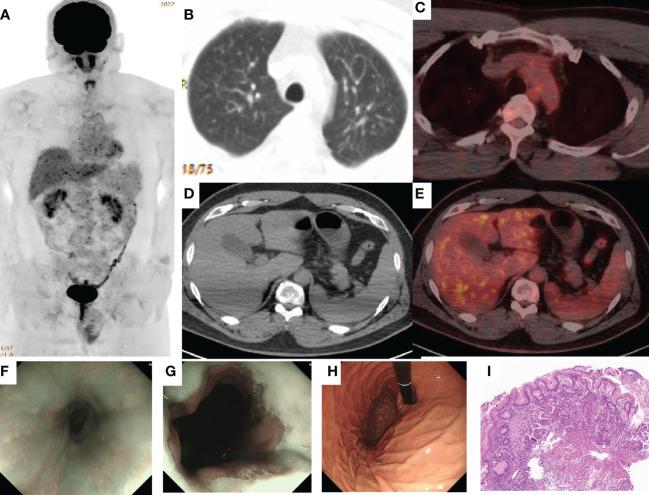
Complete response (CR) was confirmed by PET/CT and the biopsy specimen from gastroscopy. **(A–E)** 18F-2-fluoro-2-deoxy-D-glucose positron emission tomography (FDG-PET) imaging shows the best overall response of CR to treatment with AK104. FDG-avidity was abolished in the gastroesophageal junction, liver, lung, and multiple lymph nodes. **(F–H)** Gastroscopy showed that the dentate line was seen 40 cm away from the incisor, and the mucosa was slightly rough without stenosis, which was biopsied at 12 o’clock. **(I)**. H&E: There was infiltration of inflammatory cells in the superficial layer of the cardia mucosa, while the glands in the deep layer were normal and no cancer cells were found (H&E, ×100 original magnification).

**Figure 4:**
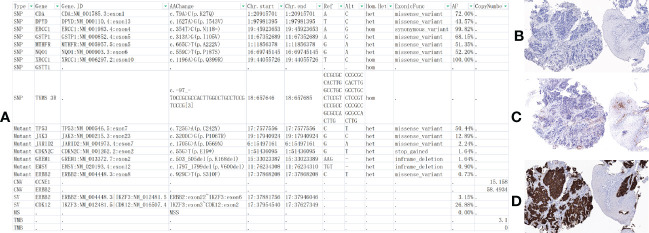
At the latest follow-up, next-generation sequencing (NGS) and immunohistochemistry (IHC) were performed on the patients’ first biopsy tissue. **(A)** NGS-Geneseeq PRIME (425-Cancer Gene Panel) of biopsy tissue to guide subsequent therapy at a recent follow-up visit. The results indicated that TP53, JAK3, JARID2, CDKN2C, GREM1, EMSY, ERBB2 mutations; copy number 58.4934 (HER-2), 15.158 (CCNE1); structural variation (SV) ERBB2, CDK12; tumor mutational burden (TMB) = 3.1, microsatellite stability (MSS). **(B–D)** IHC: EBV (−), PD-L1 Combined Positive Score, CPS = 3 (PD-L1,22C3), HER-2 (3+) (×100 original magnification). *At position 55, base G changes to T, causing a codon that should have been translated to E(glutamic acid) to become a stop codon, resulting in translation termination.

A cycle of oxaliplatin 85 mg/m^2^ d1 + capecitabine 1,000 mg/m^2^ d1–10/Q14d 1 cycle was initiated on 14 January 2021. AK104 6 mg/kg d1 + capecitabine 1,000 mg/m^2^ d1–10/Q14d regimen maintenance treatment commenced on 4 February 2021. During the CT evaluation, his condition was sustained at PR on 2 September 2021 monitoring of liver function: ALT 173.7 U/L and AST 148.4 U/L (**Figure 2C**). We delivered liver preservation therapy and, on 3 September 2021, retest liver function: ALT 189.0 U/L and AST 114.8 U/L. At this time, oxaliplatin had been discontinued for 7 months, so it was considered that liver damage was likely to be related to immunity. We gave MPSS 1 mg/kg combined with liver protection and gallbladder therapy to improve the liver function test on 14 September 2021: ALT 69.4 U/L and AST 26.5 U/L. Then the patient was treated at home with oral prednisone, and liver function returned to normal after regular review. 5 October 2021: ALT 650.4 U/L, AST 499.6 U/L, TBil 35 umol/L, DBil 23.1 umol/L, I-Bil 11.9 umol/L. Incorporating the patient’s symptoms and hematologic findings, we diagnosed grade 3 immune-mediated hepatitis. MPSS 2 mg/kg combined with liver protection and gallbladder treatment was used to improve immune hepatitis. 14 October 2021: ALT 153.6 U/L, AST 35.2 U/L. 18 October 2021: ALT 171.3 U/L, AST 41.2 U/L. Considering corticosteroid resistance in patients, we treated them with the incorporation of mycophenolate mofetil. 25 October 2021: ALT 84.7 U/L↑, AST 18.2 U/L, TBil 17.3 umol/L↑, DBil 7.3 umol/L. The patient is getting better right now. Nevertheless, the patient was excluded from the clinical study due to the long-term absence of medication. Antitumor therapy was also discontinued in view of the adverse immune response of the patient. The patient did not receive subsequent immune antitumor therapy, and immune-related hepatitis still occurred intermittently, but the disease evaluation was maintained at PR. CR was confirmed by FDG-PET and the biopsy specimen from gastroscopy on 10 June 2020 (**Figures 3A–I**). Next-generation sequencing (NGS)-Geneseeq PRIME (425-Cancer Gene Panel) of first biopsy tissue to guide subsequent therapy at a recent follow-up visit. The results indicated that TP53, JAK3, JARID2, CDKN2C, GREM1, EMSY, ERBB2 mutations; copy number 58.4934 (ERBB2), 15.158 (CCNE1); structural variation (ERBB2, CDK12); tumor mutational burden (TMB) = 3.1, microsatellite stability (MSS) (**Figure 4A**, **Supplementary Figure S1**). Immunohistochemistry (IHC): EBV (−), PD-L1 CPS = 3, HER-2 (3+) (**Figures 4B–D**).

## Discussion

Gastric cancer is a heterogeneous disease, and HER-2-positive patients have heterogeneous responses to current standard therapies. One of the key reasons for this is insufficient attention to the underlying molecular mechanisms that lead to differences in cancer aggressiveness and treatment outcomes (11). Several studies have confirmed that anti-HER-2 effects involve antibody-dependent cell-mediated cytotoxicity by immune mechanisms superior to intracellular signaling (6). In two cancer models in immunocompetent mice, recruitment or downregulation of macrophages and NK cells (the primary effector cells of Ab-dependent cellular cytotoxicity) blocked trastuzumab’s effect on tumor control. Ab-dependent cellular cytotoxicity (ADCP) and Ab-dependent cellular phagocytosis (ADCC) were validated as novel mechanisms of action of trastuzumab. It is proposed that activation of macrophages and NK cells can strengthen the anti-cancer efficacy of trastuzumab and other Ab immunotherapies by enhancing ADCP and ADCC, demonstrating that targeted effects are secondary to immune effects (12, 13). Interim data for the phase III KEYNOTE-811 trial (NCT03615326) have been published (9). The objective response rate (a secondary end point) in the first 264 patient incidents was 74.4% in the pembrolizumab group and 51.9% in the placebo group (P = 0.00006), and complete responses were more frequent (11.3% versus 3.1%). The result of the trial is still unknown, but the combination of animal experiments and the current results suggests that the immune effects of anti-HER-2 therapy can be better understood.

**Figure 5:**
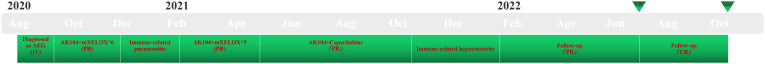
Timelines of events.

For this patient, participating in AK104 combined with chemotherapy is both an opportunity and a challenge. If the HER-2 positive status was known in advance, this patient would not have been able to participate in this trial, and the current standard anti-HER-2 treatment would have been applied, and he might not have achieved CR. By a stroke of luck, the shackles of guidelines can be broken, and the innovative application of excluding anti-HER-2 therapy can achieve this amazing clinical effect (**Figure 5**). The rapid development of antineoplastic drugs has greatly altered the way in which cancer is treated. At present, there are more and more ways to treat tumors, and many of them are too complicated. The therapeutic effect is not significantly improved, but adverse reactions are considerably increased. The aim of our therapy is to cure the disease rather than complicate its treatment. Cross-border competition–competitors are not from the same industry. Just as in the days of the horse-drawn carriage, people were looking for a faster horse, but even a faster horse could not beat the later invention of the automobile. Understanding something from other cognitive dimensions often opens the problem-solving landscape. For HER-2 positive patients, PD-1/CTLA-4 bispecific therapy has a good effect on MSI-H/dMMR population like PD-1/PD-L1 (14), and then achieve curve overtaking and lane change acceleration, bringing new first-line treatment options for more patients with positive HER-2 amplification.

## Conclusions

In short, patients with HER-2-positive advanced GEJ cancer received PD-1/CTLA-4 bispecific immunotherapy combined with chemotherapy and achieved complete remission. The simplest and most effective treatment is the best regimen. It provides a framework for future clinical and translational research of [TP53, JAK3, JARID2, CDKN2C, GREM1, EMSY, ERBB2 mutations; copy number 58.4934 (ERBB2), 15.158 (CCNE1); structural variation (ERBB2, CDK12); IHC: HER-2 (3+), EBV (−), TMB-L (3.1), MSS] subtype gastric cancer. This case illustrates the clinical benefits of this regimen, which may become a first-line therapy option for HER-2-positive patients, but further clinical trials are needed to confirm this.

## Data availability statement

The original contributions presented in the study are included in the article/**Supplementary Materials**, further inquiries can be directed to the corresponding author/s.

## Ethics statement

The studies involving human participants were reviewed and approved by the Medical Ethics Committee of the Shandong Cancer Hospital and Institute, Shandong First Medical University, and Shandong Academy of Medical Sciences. The patients/participants provided their written informed consent to participate in this study.

## Author contributions

JP, QZ, and YL analyzed and interpreted the patient data regarding the disease and the diagnosis. ZP performed the histological examination and diagnosis. JP, ZC, and BL dealt with the therapeutic management of the patient. All authors read and approved the final manuscript.
