# Supplementary figures and images for: Patients with positive HER-2 amplification advanced gastroesophageal junction cancer achieved complete response with combined chemotherapy of AK104/cadonilimab (PD-1/CTLA-4 bispecific): A case report
Source: Front Immunol. 2022 Dec 8;13:1049518. doi: 10.3389/fimmu.2022.1049518 (PMC9773983; doi:10.3389/fimmu.2022.1049518)

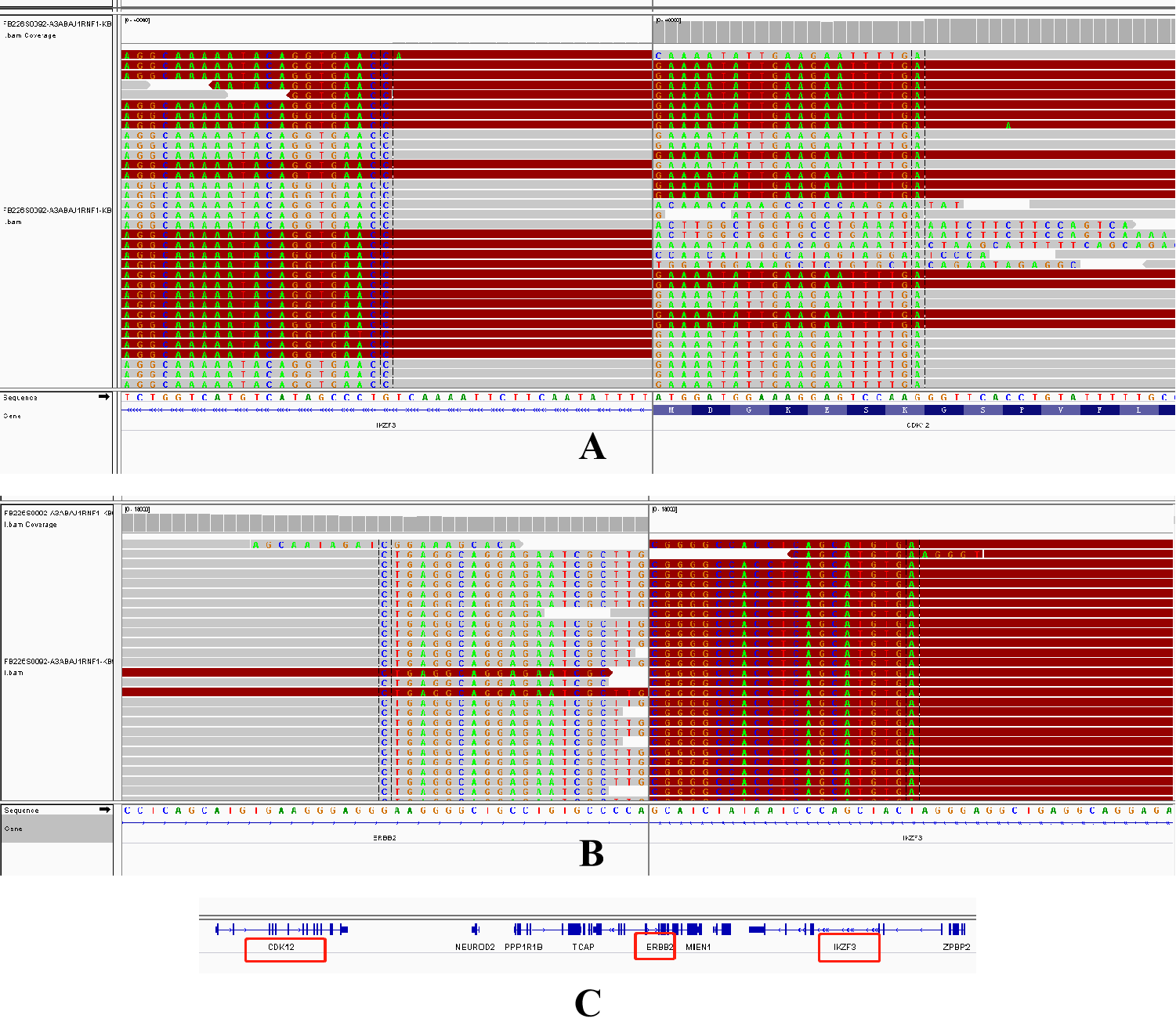

Supplement: Supplementary file 1 [file Image_1.tif]
